# A Novel Splice-Site Mutation in *MSH2* Is Associated With the Development of Lynch Syndrome

**DOI:** 10.3389/fonc.2020.00983

**Published:** 2020-06-19

**Authors:** Juyi Li, Yuanyuan Li, Haichun Ni, Zhibin Yang, Jian Chen, Yarong Li, Sheng Ding, Xiaowan Jiang, Mengjie Wang, Li Li, Xiaoyu Lv, Xiaoyun Ruan, Qian Jiang, Zhang Lei, Yong Cheng, Juan Huang, Aiping Deng

**Affiliations:** ^1^Department of Pharmacy, The Central Hospital of Wuhan, Tongji Medical College, Huazhong University of Science and Technology, Wuhan, China; ^2^Department of Pathology, The Central Hospital of Wuhan, Tongji Medical College, Huazhong University of Science and Technology, Wuhan, China; ^3^Department of Information, The Central Hospital of Wuhan, Tongji Medical College, Huazhong University of Science and Technology, Wuhan, China; ^4^Department of Endocrinology, The Central Hospital of Wuhan, Tongji Medical College, Huazhong University of Science and Technology, Wuhan, China; ^5^Department of Oncology, The Central Hospital of Wuhan, Tongji Medical College, Huazhong University of Science and Technology, Wuhan, China; ^6^Department of Gastrointestinal Surgery, The Central Hospital of Wuhan, Tongji Medical College, Huazhong University of Science and Technology, Wuhan, China; ^7^Department of Personnel, The Central Hospital of Wuhan, Tongji Medical College, Huazhong University of Science and Technology, Wuhan, China

**Keywords:** Lynch syndrome, hereditary non-polyposis colorectal cancer, *MSH2*, microsatellite instability, aberrant splicing, genetic counseling

## Abstract

Lynch syndrome (LS) is an inherited autosomal dominant disorder caused by germline mutations of mismatch repair (MMR) genes, including *MSH2, MSH6, PMS2*, and *MLH1*. This study aimed to analyze the molecular defects and clinical manifestations of an affected family and propose appropriate individual prevention strategies for all mutation carriers. A novel splicing mutation (c.1661+2 T>G) was identified in the *MSH2* gene, which was found to co-segregate among affected family members by Whole exome sequencing (WES). RT-PCR analysis confirmed that c.1661+2 T>G could produce 3 transcripts, including 1 normal transcript and 2 aberrant transcripts. The 2 aberrant transcripts resulted in premature termination at the 6th nucleotide codon of *MSH2* exon 11, so that the predicted products of the mutant *MSH2* mRNAs were truncated proteins of 505 amino acids (with all of exon 10 deleted) and 528 amino acids (with a deletion of 82-nucleotides in exon 10), resulting in the loss of the interaction domain, the ATP domain and post-translationally modified residues. Quantitative RT-PCR (qRT-PCR) analysis showed that *MSH2* mRNA levels in all patients were reduced to only 1/4 of the control levels. Our study reveals that a novel splicing mutation (c.1661+2 T>G) in the *MSH2* gene causes LS and reaffirms the importance of genetic testing for LS.

## Introduction

Lynch Syndrome (LS) is a hereditary autosomal dominant disorder associated with a higher risk of colorectal cancer (CRC) and other epithelial malignancies. It accounts for ~2–3% of all CRC cases diagnosed annually (1). Also known as hereditary nonpolyposis colorectal cancer (HNPCC), LS is associated with germline mutations in one (or more) genes (*MLH1, MSH2, MSH6*, and *PMS2*) associated with mismatch repair (MMR), as well as with deletions in the *EpCAM* gene that lead to the loss of MSH2 expression (2, 3). Patients with LS have a lifetime risk of CRC of 52–82% and of gastric cancer of 6–13%. They also show an increased risk of suffering from cancer of the liver, urinary tract, small intestine, gallbladder duct, pancreas and brain, as well as increased risk of endometrial (25–60%) and ovarian cancer (4–12%) in females (4). Mutations in these genes disrupt mismatch repair, leading to genome instability and faster cancer progression. Therefore, individuals with mutations in these genes are more likely to develop cancer than the general population and often develop cancer earlier.

LS is associated with 2–3% of all CRC cases, which proves that all CRC tumors should be screened for mismatch repair defects through microsatellite instability (MSI) tests or immunohistochemistry for DNA MMR proteins (5, 6). Around 90% of LS cases are caused by *MLH1* and *MSH2* mutations, while about 10% of LS patients carry *MSH6* and *PMS2* mutations (7). When patients meeting Amsterdam II or Bethesda clinical criteria are diagnosed through molecular analysis, this information is useful for the entire family. Periodic health checks are recommended for family members carrying the same variants (8).

Therefore, it is important to identify disease-causing mutations in these patients to guide the clinical management of the family, to provide genetic counseling and for pre-symptomatic monitoring (9, 10). Here we report a novel *MSH2* splice-site mutation (c.1661+2 T>G) in a Chinese family with LS. The purpose of this study was to analyze the molecular defects and clinical manifestations in this family, in order to provide appropriate individual prevention strategies for all mutation carriers.

## Materials and Methods

### Patients

This study was approved by the Ethics Committee of the Central Hospital of Wuhan. Informed consent was obtained from all participants. The proband and her parents, who were successively diagnosed with hereditary non-polyposis colorectal cancer and underwent partial colon resection, were recruited from the Department of Gastrointestinal Surgery at the Central Hospital of Wuhan.

### Mismatch Repair Protein Immunohistochemistry

Regular 5 μm, paraffin-embedded tissue sections were used to detect the expression of 4 mismatch repair proteins (*MLH1, PMS2, MSH2*, and *MSH6*). Briefly, slides were incubated overnight with the primary antibodies (anti-MLH1, mouse monoclonal antibody, clone ES05; anti-PMS2, rabbit monoclonal antibody, clone EP51; anti-MSH2, mouse monoclonal antibody, clone FE11; anti-MSH6, rabbit monoclonal antibody, clone EP49) (Jiayuan Biomedical Engineering Co., Ltd, Wuhan, China) (11).

### Microsatellite Instability Test

DNA samples were isolated from the tumors. We subsequently performed multiplex PCR analysis at five microsatellite repeat loci (BAT-25, BAT-26, MONO-27, CAT-25, and NR-24), as previously described (12).

### Whole-Exome Sequencing for Variant Identification

Genomic DNA was extracted from the peripheral blood of the proband. DNA fragments were sequenced using a high-throughput sequencer (Illumina HiSeq 2500 Analyzer, Illumina; CA, United States). Single nucleotide variant and insertion and deletion queries were performed as previously described (11, 13). The reference genome for whole-exome sequencing was UCSC hg19, NCBI build 37.

### Functional Impact of Disease-Related Variants

Integrated mutation prediction software (SIFT, PolyPhen2, Mutation Taster, Mutation Assessor, FATHMM, GERP plus, PhyloP100, and PhastCons100) packages were used for analyses of the identified variants (14, 15). The possible influence of a mutation in an intron was evaluated based on the bioinformatic splicing tool Human Splicing Finder (HSF) version 3.0 (http://www.umd.be/HSF3/).

### Sanger Sequencing

Polymerase chain reaction (PCR) amplification and Sanger sequencing were used to verify the results of high throughput sequencing.

Furthermore, Sanger sequencing of unrelated healthy controls was carried out at the individualized medical laboratory of the Central Hospital of Wuhan (*n* = 200) to confirm the molecular diagnosis.

### PCR Analysis

Reverse transcription-polymerase chain reaction (RT-PCR) and quantitative RT-PCR (qRT-PCR) were performed to detect *MSH2* transcript variants in peripheral blood cells of patients and healthy volunteers. Total RNA was extracted using the TRIzol method (RNA extraction kit, Invitrogen). First-strand cDNA synthesis was performed using a reverse transcription kit (Thermo). The primer pair sequences for *MSH2* analysis by RT-PCR (nested PCR) were as follows: RT710-1F: GTGGAAAACCATGAATTCCTTGTA and RT710-619R: CAGTAATGATGTGGAACATCTGTTTAT; RT710-19F: CTTGTAAAACCTTCATTTGATCCTAA and RT710-583R: AGTATACGTCATTAGGAATAAATGCAAT. The PCR products were verified by Sanger sequencing. The primer pair sequences for quantification of *MSH2* expression by qRT-PCR were as follows: Forward: AGTCTCCACGTTCATGGCTG; Reverse: TCAGTGGTGAGTGCTGTGAC. GAPDH was used as the internal control.

### Analysis of Functional Domains of Mutant Proteins

The functional domains were visualized using an online website [UniProtKB-P43246 (MSH2_HUMAN), https://www.uniprot.org/uniprot/P43246/protvista], to analyze the effects of the mutations.

## Results

### Family Characteristics

**Figure 1:**
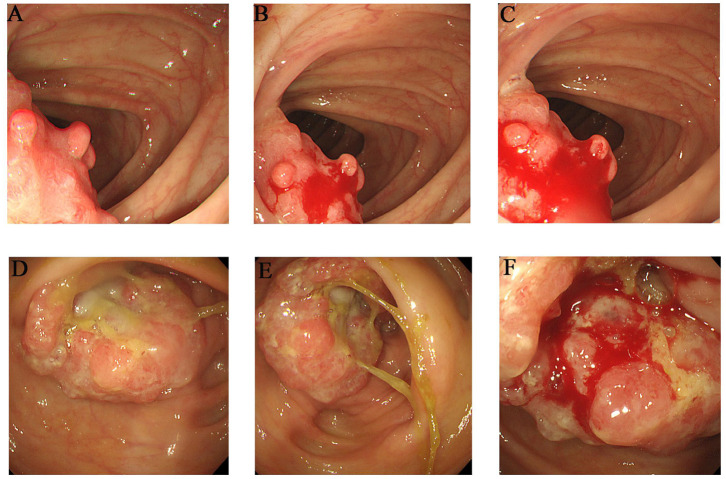
Clinical description. Colonoscopies. **(A–C)** Transverse colon, with a neoplasm measuring 2^*^2 cm (III-1). **(D–F)** Ascending colon, showing an irregularity- about 3.0 cm of elevated mucosa (II-1).

The proband (III-1) was a 55-year-old female who had undergone a partial transverse colon resection due to a poorly differentiated adenocarcinoma. The proband's father (II-2) and mother (II-1) were diagnosed with colorectal cancer at the ages of 39 and 81 years, respectively, and had undergone partial ascending colon and cecum resection. Furthermore, the proband's grandmother (I-4) died of colon cancer, although the clinical details were not clear. Members II-3, II-4, and II-5 are 75–90 years old, and are in good physical condition with no history of tumors. The detailed pedigree is shown in Supplementary Figure 1. The colonoscopies of affected family members (II-1 and III-1) are shown in Figure 1.

### Immunohistochemical Analysis

**Figure 2:**
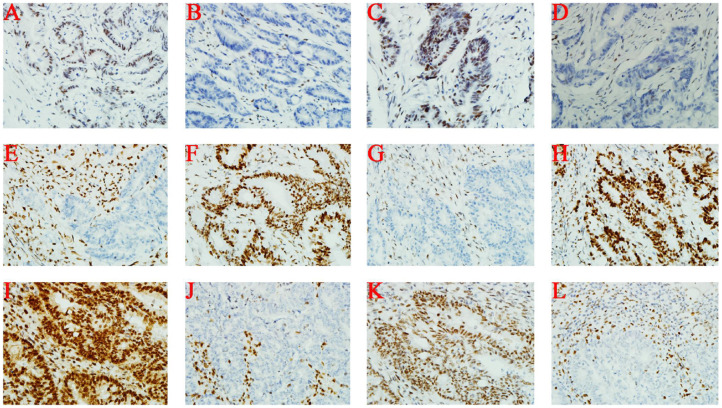
Immunohistochemistry. **(A–D)** III-1; **(E–H)** II-1; **(I–L)** II-2. From left to right, the antibodies in each column were specific for MLH1, MSH2, PMS2, and MSH6.

Immunohistochemical staining of tumor cells (III-1 and II-2) demonstrated moderate positivity for MLH1 (Figures 2A,I) and PMS2 (Figures 2C,K) proteins, weak positivity for MSH6 (Figures 2D,L), and lack of expression of MSH2 protein (Figures 2B,J). On the other hand, MSH2 (Figure 2F) and MSH6 (Figure 2H) proteins were moderately expressed, and MLH1 (Figure 2E) and PMS2 (Figure 2G) proteins were not expressed at all in tumor cells from II-1.

### Microsatellite Instability (MSI) Analysis of Tumor Tissues

In the proband's tumor tissue, high MSI was observed at BAT-25, MONO-27, CAT-25, BAT-26, and NR-24 mononucleotide markers. Based on the immunohistochemistry analysis of mismatch repair proteins conducted in the proband, we conclude that the mutation interfered with the gene mismatch repair function of the MSH2 protein, which in turn caused microsatellite instability.

### Genetic Test Results

Whole-exome sequencing data is summarized in Supplementary Table 1. A total of 121,795 mutations were found, including 104,413 SNPs and 17,382 InDels. The number of synonymous mutations, nonsense mutations, missense mutations, new SNPs and new InDels were 10965, 89, 10376, 486, and 865, respectively. Further analysis showed there were 17, 15, 2, and 7 known mutations in *PMS2, MSH2, MLH1*, and *MSH6*, respectively, and that their mutation frequencies were >1%; therefore, these mutations were excluded as pathogenic mutations. In contrast, a novel mutation (NM_000251.2: c.1661+2T>G) was identified in *MSH2* which was deleterious to protein function, was strongly associated with colorectal cancer and had not been previously reported. In this regard, a known mutation (NM_000251.2: c.1661+2T>C, rs1553366680, likely pathogenic, VCV000455513.2) in the same *MSH2* site reportedly caused aberrant splicing. Therefore, we considered the (NM_000251.2: c.1661+2T>G) mutation in *MSH2* as pathogenic.

We performed Sanger sequencing to detect the (c.1661+2T>G) *MSH2* mutation in members of the patient's family in order to provide genetic counseling. Analysis showed that the germline mutation in *MSH2* was also carried by the proband's father (II-2) and daughter (IV-1), but not by the proband's mother (II-1) or brother (III-2, in good health). Furthermore, the proband's grandmother (I-4) died of colon cancer, so these results suggested that the germline mutation (c.1661+2T>G) in *MSH2* was pathogenic, and was inherited from the proband's father (II-2) and grandmother (I-4). The proband's mother (II-1) may have developed colorectal cancer by coincidence, not by inheritance, whereas the proband's daughter (IV-1) carried the mutation and had slight gastrointestinal symptoms. Importantly, no (c.1661+2T>G) mutation in the *MSH2* gene was found in any of the 200 unrelated healthy controls, based on Sanger sequencing (Supplementary Figure 2).

### *In silico* Splicing Analysis

Splicing mutations can be identified by HSF, allowing a better understanding of the clinical and biological data. The HSF test showed that the c.1661+2T>G mutation in intron 10 resulted in the loss of the splice site and possible activation of novel splice sites in exon 10.

### Splicing Defect in *MSH2* c.1661+2T>G

The novel c.1661+2T>G mutation was located at the junction of exon 10 and intron 10. It was considered to be related to alternative splicing and was identified as pathogenic based on MutationTaster analysis. RT-PCR was used to characterize abnormal splicing patterns in this genomic region, including exons 9, 10, 11, and 12.

**Figure 3:**
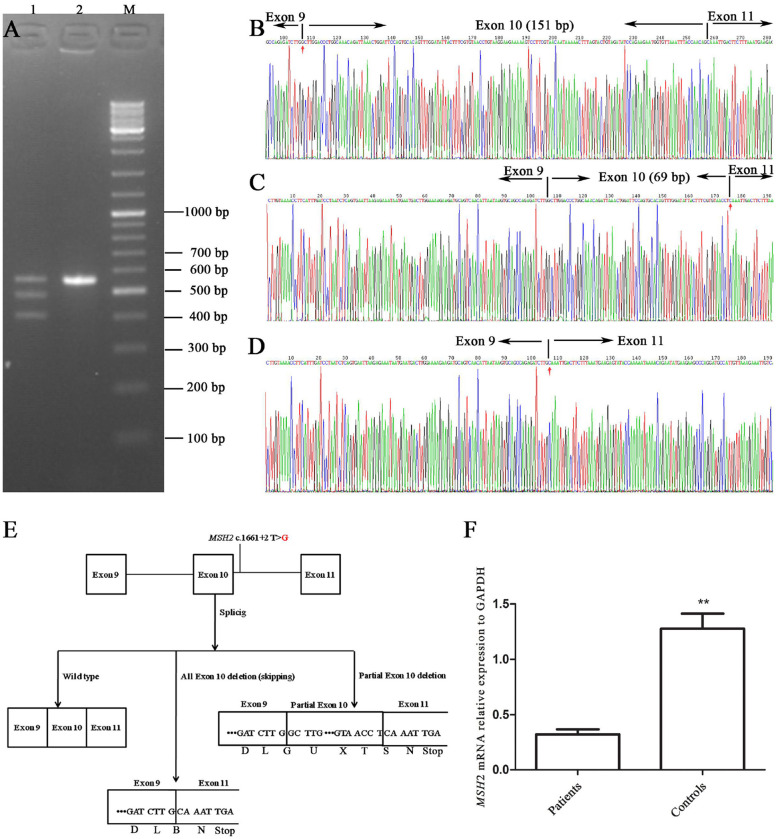
Alternative splicing of *MSH*2 c.1661+2T>G. **(A)** RT-PCR of genomic region encompassing exons 9, 10, 11, and 12 to characterize the abnormal splicing; 1: patient III-1; 2: control III-2; M: markers. **(B–D)** Sanger sequencing analysis of alternatively spliced products; **(B)** wild type; **(C)** 82-nucleotide deletion in exon 10; **(D)** entire exon 10 deletion. **(E)** Schematic representation of splicing models. **(F)** qRT-PCR analysis of mutation carriers (II-2, III-1, and IV-1) and controls (II-1, III-2 and another healthy volunteer). Results are expressed as the mean ± SE (**P < 0.01).

The RT-PCR products of the healthy control (III-2) and the proband were analyzed by gel electrophoresis. A_565 bp fragment (expected wild type) plus two additional smaller fragments of _483 bp and _414 bp (Figure 3A) were seen, corresponding to abnormal DNA fragments caused by rare splicing of *MSH2* c.1661+2T>G. Sequencing of the RT-PCR products revealed (Figure 3B) an 82-nucleotide deletion in exon 10 (Figure 3C) and an entire exon 10 deletion in the *MSH2* cDNA (Figure 3D), which were consistent with the size of the RT-PCR products of the patient's DNA (Figure 3A). The 2 deletions caused premature termination at the TGA codon in the 6th nucleotide upstream of *MSH2* exon 11, resulting in a truncated protein of 505 amino acid residues (all exon 10 deletion) or 528 amino acid residues (82-nucleotide deletion in exon 10), whereas the *MSH2* wild-type protein has 934 amino acid residues (Figure 3E).

The qRT-PCR results showed that *MSH2* mRNA levels in patients were only 1/4 those of controls (Figure 3F). These results suggest that mutant *MSH2* mRNA is insufficient, resulting in deficient MSH2 protein expression in patients.

### Analysis of the Mutant Proteins

**Figure 4:**
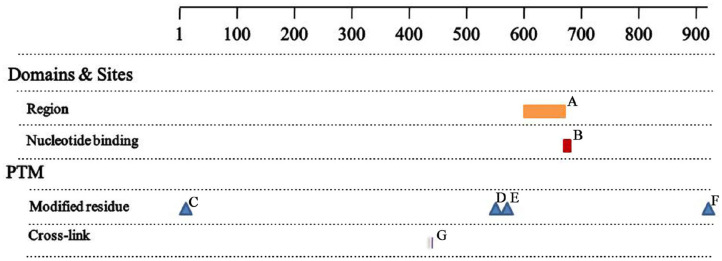
Analysis of the mutant proteins **(A)** Region 601-671, interaction with EXO1; **(B)** NP_BIND 669-676, ATP; **(C)** MOD_RES 2-2, N-acetylalanine; **(D)** MOD_RES 555-555, N6_acetyllysine; **(E)** MOD_RES 567-567, N6_acetyllysine; **(F)** MOD_RES 921-921, phosphoserine; **(G)** CROSSLINK 430-430, Glycyl lysine isopeptide (Lys-Gly) (interchain with G-Cter in SUMO2).

The *MSH2* c.1661+2T>G mutation produced 2 truncated proteins of 505 and 528 amino acid residues. Based on sequence alignment, this resulted in the loss of the region of interaction with EXO1 (residues 601–671, Figure 4A), the nucleotide ATP binding region (residues 669–676, Figure 4B), and three post-translationally modified residues, including two N6-acetyllysines (residues 555 and 567, Figures 4D,E) and one phosphoserine (residue 921, Figure 4F). There was no change in the two functional sites (Figures 4C,G) between wild-type and mutant proteins.

## Discussion

In this study, a novel splicing mutation (c.1661+2 T>G) was identified in the *MSH2* gene of a typical LS/HNPCC family. This mutation was not found in 200 healthy controls. The *MSH2* mutation generated 2 truncated proteins with loss of domains and binding sites and mutant *MSH2* mRNA levels were insufficient. Therefore, this mutation is believed to be associated with the disease. To our knowledge, this is the first report of an (c.1661+2 T>G) *MSH2* mutation associated with LS.

Consistent with prior studies, defects in MMR protein function affected the immunohistochemistry staining patterns, suggesting which genes were damaged. Weak or no staining of MLH1 and PMS2 proteins indicate *MLH1* pathogenic variants; weak or no staining of MSH2 and MSH6 proteins indicate *MSH2* pathogenic variants; whereas loss of MSH6 or PMS2 proteins indicate pathogenic variants of the corresponding genes (16). Our immunohistochemistry results (loss of MSH2 and MSH6 proteins) were in agreement with the molecular findings (a pathogenic variant of *MSH2*), supporting the notion that MMR protein losses were caused by a pathogenic mutation of the corresponding MMR genes.

In this study, the MSH2 and MSH6 proteins were moderately expressed, and the MLH1 and PMS2 proteins were not expressed in tumor cells from (II-1). However, the above result was opposite to that of patients II-3 and III-1. In addition, no harmful mutations of *MLH1* and *PMS2* were found in patient III-1. Therefore, it was concluded that the pathogenic mutation was inherited from the proband's father (II-2), whereas it was an accident that the proband's mother (II-1) also had CRC. However, the proband's daughter (IV-1) carried the pathogenic mutation and had slight gastrointestinal symptoms, so she should be examined regularly with an enteroscope. If a polyp is found, it should be promptly removed to prevent cancer. In addition, mutation carriers can consider undergoing prophylactic subtotal colectomy, rather than traditional segmentectomy, as it increases the risk of secondary colectomy (17).

*MSH2* germline mutations associated with LS mainly cause truncations of the MSH2 protein, but they may also be single amino acid substitutions (20–25%) (18). ATP binding and hydrolysis play a key role during mismatch repair. MutS α (MSH2-MSH6 heterodimer) -associated ATPase activity functions like a molecular switch to regulate binding: mismatched DNA triggers ADP → ATP exchange, resulting in a recognizable conformational transition (19). However, the *MSH2* c.1661+2T>G mutation resulted in the loss of ATP binding sites, loss of ATP regulation, loss of DNA mismatch repair and cancer progression. To date, more than 5,000 and 100 variants of the *MSH2* gene have been reported in the World and China, respectively. Recently, some studies reported that *MSH2* mutation carriers were more prone to develop extracolonic cancers or multiple tumors, while carriers of *MSH6* mutations had a higher frequency of endometrial cancer (18). Therefore, genotype-phenotype correlation studies will permit more specific treatments for patients with LS.

Genetic testing of MMR genes has been widely used for the diagnosis of LS (20). MMR genes can correct DNA replication errors. DNA replication errors can lead to a progressive accumulation of mutations in cells and eventually to cancer (6). LS patients benefit from increased surveillance and it is important to identify this syndrome in patients and their family members. Genetic consultation and regular follow-up should be carried out to guide individualized treatment of cancer-afflicted families with MMR gene expression deficiency.

## Limitations

This study has some limitations. Only one family with LS was included in this study, so more families are needed to confirm our findings. The precise molecular mechanism by which the novel splicing mutation (c.1661+2 T>G) in the *MSH2* gene leads to the development of LS needs to be investigated in the future.

## Conclusions

To our knowledge, this is the first study to show that a novel splicing mutation (c.1661+2 T>G) in the *MSH2* gene causes LS, reaffirming the importance of genetic testing for this condition. This novel finding expands the database of germline mutations of the *MSH2* gene and the scientific basis for accurate diagnosis and treatment of LS.

## Data Availability Statement

The raw data supporting the conclusions of this article will be made available by the authors, without undue reservation, to any qualified researcher.

## Ethics Statement

The studies involving human participants were reviewed and approved by the Ethics Committee of The Central Hospital of Wuhan. The patients/participants provided their written informed consent to participate in this study.

## Author Contributions

AD and YC: conceived and designed the experiments. JL and YuL: performed the experiments. JL, HN, ZY, JC, YaL, SD, XJ, MW, LL, XL, XR, QJ and JH: analyzed the data. JL and AD: wrote the paper. All authors contributed to the article and approved the submitted version.

## Conflict of Interest

The authors declare that the research was conducted in the absence of any commercial or financial relationships that could be construed as a potential conflict of interest.

## Supplementary Material

Supplementary Figure 1 Family pedigree of the patient. squares, males; circles, females; slashs, died; black symbols, colorectal cancer, the proband (III-1).

Supplementary Figure 2 Electropherogram of the Sanger sequencing. **(A)** TT wild type; **(B)** TG heterozygote type.

Supplementary Table 1 Proband's whole-exome sequencing in detail.

## Abbreviations

LS: Lynch syndrome
MMR: mutations in mismatch repair
WES: Whole exome sequencing
PCR: Polymerase chain reaction
RT-PCR: reverse transcription-polymerase chain reaction
qRT-PCR: quantitative RT-PCR
CRC: colorectal cancer
HNPCC: hereditary nonpolyposis colorectal cancer
HSF: Human Splicing Finder.
