# Supplementary figures and images for: A Novel Splice-Site Mutation in MSH2 Is Associated With the Development of Lynch Syndrome
Source: Front Oncol. 2020 Jun 19;10:983. doi: 10.3389/fonc.2020.00983 (PMC7318799; doi:10.3389/fonc.2020.00983)

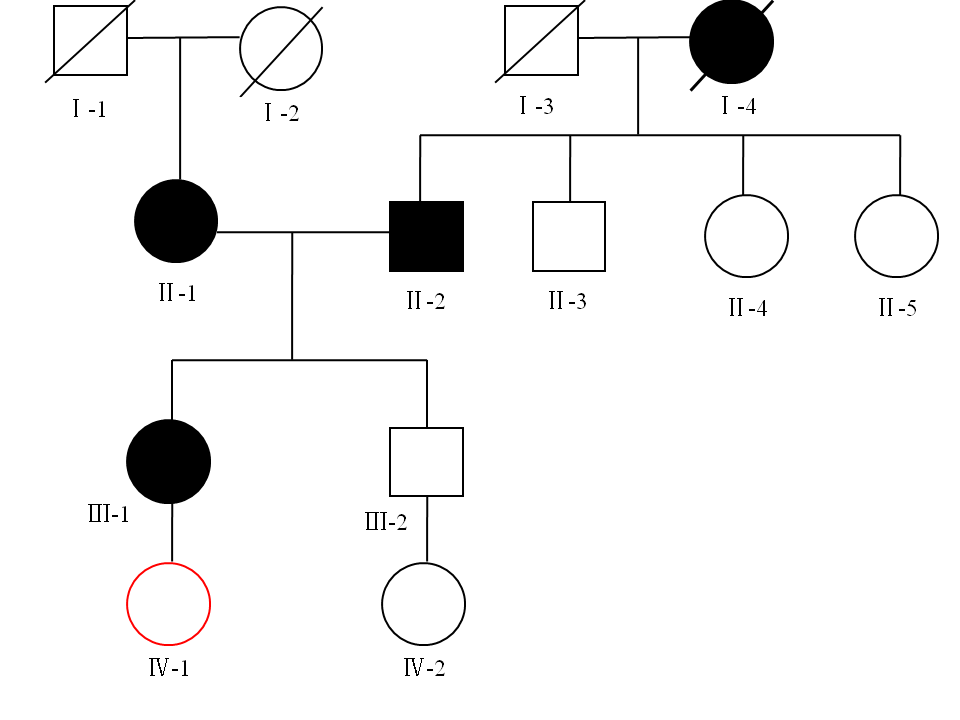

Supplement: Supplementary Figure 1 — Family pedigree of the patient. squares, males; circles, females; slashs, died; black symbols, colorectal cancer, the proband (III-1). [file Image_1.tif]

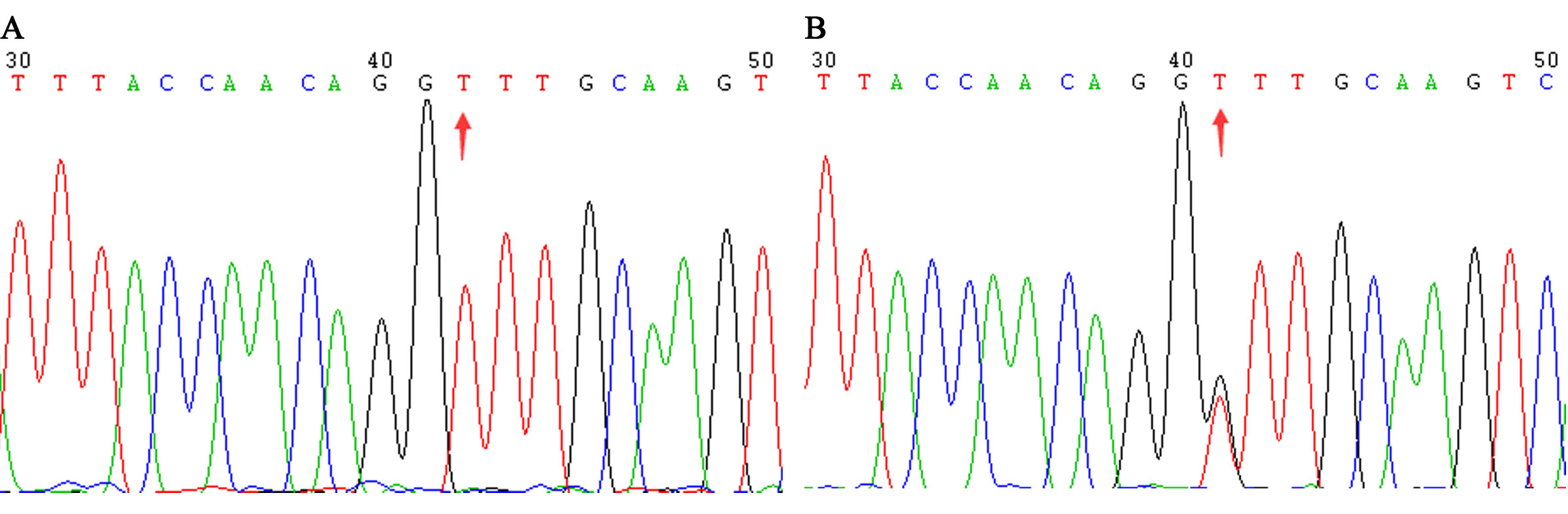

Supplement: Supplementary Figure 2 — Electropherogram of the Sanger sequencing. (A) TT wild type; (B) TG heterozygote type. [file Image_2.jpg]
