# Supplementary material for: A Novel Splice-Site Mutation in MSH2 Is Associated With the Development of Lynch Syndrome
Source: Front Oncol. 2020 Jun 19;10:983. doi: 10.3389/fonc.2020.00983 (PMC7318799; doi:10.3389/fonc.2020.00983)
Supplement: Supplementary Table 1 — Proband's whole-exome sequencing in detail. [file Table_1.pdf]

Supplementary Table 1. Proband's whole-exome sequencing in detail

**Exome Capture Statistics**	**Proband**
Raw reads (bp)	229789072
Clean reads (bp)	229158346
GC (%)	52.09
Initial bases on target (bp)	60456963
Total effective reads (bp)	201325227
Total effective bases (Mb)	19884.90
Effective sequences on target (Mb)	14486.50
Capture specificity (%)	72.85
Mapping rate on genome (%)	99.91
Duplicate rate on genome (%)	12.16
Mismatch rate in target region (%)	0.42
Average sequence depth on target (X)	239.62
Fraction of target region covered >=1X (%)	99.71
Fraction of target region covered >=4X (%)	99.66
Gender	Female
